# 

**Published:** 1919-01

**Authors:** 


### CONTENTS FOR THE JANUARY ISSUE

EDITORIALS.

For This Good Year.............................................. 223
Create Healthy Bodies.............................................. 224
Ventilation and Sanitation.............................................. 225
The Joy Treatment.............................................. 225
An Allied Medical Organization.............................................. 226
Is It the Chinese Plague?.............................................. 226
Read All of Them.............................................. 227
Papers on Genito-Urinary Surgery.............................................. 228
Thank You.............................................. 228

ORIGINAL ARTICLES.

Hypertrophy of the Prostrate Gland. Dr. Gustav Kolischer 230
Removal of the Eye-ball Under Local Anesthesia. Dr. Alberto G. Garcia.............................................. 235
Appendicitis Complicating Pregnacy. Aime Paul Heimeck, M. D.............................................. 238
An Appreciation of Dr. G. Henri Bogart.............................................. 243
Medical Miscellany.............................................. 246
The "Flu" Poem.............................................. 246
Prenatal Care.............................................. 246
Armenian and Syrian Relief Fund.............................................. 248
Seventy-five Hospitals at Army Camps turned over to Surgeon General.............................................. 249
News Items.............................................. 250
Deaths .............................................. 252
Personals .............................................. 253
Army News.............................................. 254
Old Jokes.............................................. 256

